# Validity and feasibility of remote measurement systems for functional movement and posture assessments in people with axial spondylarthritis

**DOI:** 10.1049/htl2.12038

**Published:** 2022-12-02

**Authors:** Erin Hannink, Maedeh Mansoubi, Neil Cronin, Benjamin Wilkins, Ali A. Najafi, Benjamin Waller, Helen Dawes

**Affiliations:** ^1^ Centre for Movement, Occupational and Rehabilitation Science (MOReS) Oxford Brookes University Oxford UK; ^2^ Oxford University Hospitals NHS Foundation Trust Oxford UK; ^3^ Intersect@Exeter, Medical School University of Exeter Exeter UK; ^4^ Neuromuscular Research Centre Faculty of Sport and Health Sciences University of Jyvaskyla Jyvaskyla Finland; ^5^ School of Sport and Exercise University of Gloucestershire Gloucestershire UK; ^6^ Good Boost Wellbeing Limited London UK; ^7^ Biomedical Research Center Medical School Faculty of Health and Life sciences University of Exeter Exeter EX1 2LU United Kingdom; ^8^ Physical Activity, Physical Education, Sport and Health Research Centre (PAPESH) Sports Science Department School of Science and Engineering Reykjavik University Reykjavik Iceland

**Keywords:** computer vision, computerised monitoring, health care, patient monitoring, patient rehabilitation

## Abstract

Introduction: This study aimed to estimate the criterion validity of functional movement and posture measurement using remote technology systems in people with and without Axial spondylarthritis (axSpA).

Methods: Validity and agreement of the remote‐technology measurement of functional movement and posture were tested cross‐sectionally and compared to a standard clinical measurement by a physiotherapist. The feasibility of remote implementation was tested in a home environment. There were two cohorts of participants: people with axSpA and people without longstanding back pain. In addition, a cost‐consequence analysis was performed.

Results: Sixty‐two participants (31 with axSPA, 53% female, age = 45(SD14), BMI = 26.6(SD4.6) completed the study. In the axSpA group, cervical rotation, lumbar flexion, lumbar side flexion, shoulder flexion, hip abduction, tragus‐to‐wall and thoracic kyphosis showed a significant moderate to strong correlation; in the non‐back pain group, the same measures showed significant correlation ranging from weak to strong.

Conclusions: Although not valid for clinical use in its current form, the remote technologies demonstrated moderate to strong correlation and agreement in most functional and postural tests measured in people with AxSA. Testing the CV‐aided system in a home environment suggests it is a safe and feasible method. Yet, validity testing in this environment still needs to be performed.

## Graphical abstract

This article introduced and evaluated the validity of a new artificial intelligence‐driven method of automatically analysing a range of standard clinical functional tests. Remote technology systems in rehabilitation have the potential to reduce health inequality and improve cost and time effectiveness for both patients and the health system. In addition to demonstrating validity in most functional and postural measures, feasibility results show that using this computer vision‐aided system in a home environment is a safe and viable method that can widen accessibility and affordability.

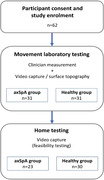

## 1 INTRODUCTION

Back pain is one of the most common health problems, and an estimated one‐third of adults in the UK are affected each year [1, 2]. One condition that causes chronic back pain is axial spondylarthritis (axSpA). This chronic inflammatory disease primarily affects spinal joints, resulting in pain and joint stiffness symptoms and altered posture. AxSpA affects approximately five in 1000 adults in the UK and is a condition that encompasses both people with ankylosing spondylitis (AS), defined by radiographic evidence of structural changes, and people with non‐radiographic axial spondyloarthritis [3]. Inflammation of the axial spine results in a clinical presentation of pain and reduced spinal mobility, which is often misdiagnosed or overlooked. Symptoms of axSpA first present as inflammatory back pain in people during the third decade of life, impacting on work, family and social commitments causing both economic and humanistic burden [4]. The clinical presentation requires both pharmacological and non‐pharmacological treatments management with regular follow‐up to optimise therapy [5].

To clinically identify the pattern and severity of reduced joint mobility, multiple tools have been developed to objectively assess these restrictions in the axSpA population. The most common non‐radiographic clinical assessment tool is the Bath Ankylosing Spondylitis Metrology Index (BASMI), an index of five simple clinical measurements to assess axial status [6]. The Edmonton Ankylosing Spondylitis Metrology Index (EDASMI) is an index of four similar clinical measurements that was developed to be more responsive to change than the BASMI yet is less widely used [7]. In further effort to increase measurement precision of the clinician‐administered BASMI and EDASMI, the University of Cordoba Ankylosing Spondylitis Metrology Index (UCOASMI) was developed to measure by automated motion capture using four cameras and 33 reflective markers placed on anatomical landmarks [8, 9]. More recently, inertial measurement unit (IMU) sensor‐based systems have been employed to measure spinal mobility using five IMUs attached along the spine [10, 11].

These tools and methods described require either a clinician for measurement or specialised equipment, for example, motion capture system or IMUs and analytic expertise. Therefore, usability and acceptability are a limitation that may prevent regular monitoring. More remote systems, for example, markerless pose estimation using computer vision, have evolved with the potential to be used directly by patients to enhance telerehabilitation [12]. Computer‐vision (CV) is a branch of artificial intelligence that can be used to automate analysis of human movement analysis from videos. By using CV‐aided methods to analyse specific functional movements captured on video, both clinicians and patients can have access to a powerful tool that could bridge the gap between the clinic and home. In addition to functional movement, postural deficits are present in people with axSpA; therefore, monitoring posture with a remote system using a surface topography tool could be important and valuable. This CV‐aided system may also have the potential to be a more cost‐effective method of evaluating and monitoring people with axSpA compared to an in‐person clinical evaluation. Remote and automated monitoring technology has the potential to work alongside the clinical team by identifying when there have been significant changes in joint mobility and posture, therefore, reducing clinician time and decreasing unnecessary travel, reducing health system pressures while at the same time creating the opportunity for more frequent access and greater accessibility to better management.

This study aimed to estimate the criterion validity of functional movement and posture measurement using remote technology systems in people with and without axSpA by comparing them to measurements performed by a trained clinician. The secondary aims were to understand the feasibility of implementing remote technology systems in the laboratory and home environments, and to estimate the cost consequences of the remote technology systems compared to a face‐to‐face clinical visit.

## 2 METHODS

### 2.1 Study design

This study was a two‐part cross‐sectional observational study. In part one, the criterion validity was measured in a movement laboratory setting with measurement by an experienced physiotherapist established as the reference test. Subsequently, in part two, the same participants captured videos in their homes for additional CV‐aided analyses, which were used to help assess the feasibility of capturing data in the home environment. The study was conducted and evaluated according to the Consensus‐based Standards for the selection of health Measurement Instruments (COSMIN) pathway for validity and reported according to the Strengthening the Reporting of Observational Studies in Epidemiology (STROBE) Statement [13, 14]. Ethical approval was granted by the University Research Ethics Committee (reference: 201429), and the study was conducted in compliance with the Declaration of Helsinki.

### 2.2 Participants

The study included men and women 18 years or older who were willing and capable of uploading videos from a smartphone or webcam. People with axSpA were recruited through the local National Axial Spondylosis Society (NASS) network, and people who reported no long‐standing back pain were recruited through social media and advertisement. Individuals were excluded from participation if they had surgery within 6 months, were unable to stand independently, were unable to pass screening questions to participate in physical activity (physical activity readiness questionnaire, PAR‐Q), had a serious neurological condition that prevented normal movement or walking ability, or had any severe medical conditions. A minimum of 17 participants were required per group (axSpA and non‐back pain groups), assuming 1‐beta = 0.90, alpha = 0.05 and effect size |ρ| = 0.50.

### 2.3 Methods of measurement

The CV‐aided system approach (Good Boost CV system, Good Boost Wellness, UK, 2021) in this study involved a modified version of OpenPose, a computer vision algorithm trained to detect key landmarks on the human body within camera images [15, 16]. For a given frame of image/video data, OpenPose returns predicted *x*, *y* coordinates for each body part and each human detected in the image. *X*, *y* coordinates were used to compute metrics such as joint angles and distances (in pixels) between two body parts for the index of movements. To translate distance values into real‐world distances, at the start of each movement, the participant or investigator held up a calibration checkerboard parallel to the camera and at the same distance at which the movement was performed; Python's OpenCV package was used to automatically detect the corners of the checkerboard to scale all distance values from pixels to centimetres [17]. The videos taken in the movement laboratory were captured by a Logitech C920 pro HD webcam (©2021 Logitech, UK) with 1080p resolution and 30 frames per second sampling rate. The videos taken in the home setting were captured by the participant's smartphone camera, tablet camera or webcam. Spinal curvature was measured in the laboratory only using a portable surface topography method employing the Microsoft Kinect sensor V2 (Microsoft Corporation, Seattle, Washington, USA) and using an established method to measure thoracic kyphosis [18]. The reference tests were a series of standard clinical assessments measured by an experienced physiotherapist who was blinded to the remote technology system analyses and results [16, 19].

### 2.4 Outcome measures for criterion validity

The index of tests used as the primary outcome measures were selected based on their relevance and representation in the BASMI and EDASMI and narrowed down after trialling all functional tests with a sample group before the study commenced. All tests and instructions were standardised. The following tests were measured by both a physiotherapist (reference test) and performed for video recording for subsequent CV‐aided analysis: lumbar side flexion, lumbar forward flexion, tragus‐to‐wall distance (TWD), cervical rotation seated, hip internal rotation, hip abduction standing, shoulder flexion and five times sit‐to‐stand (5 × STS) (Table 1). Standing posture was measured by a physiotherapist using a flexible ruler (reference test) [20, 21] and captured by the Kinect sensor. See protocol report for further detail [16].

**TABLE 1:** Description of tests

**Test**	**Brief description**
Lumbar side flexion	Active ROM test for standing lateral side flexion; distance of hand displacement measured in cm.
Lumbar forward flexion	Active ROM test for forward flexion; distance of fingertips to lateral malleolus measured in cm.
Tragus‐to‐wall	Standing global forward posture; horizontal distance from wall measured in cm.
Cervical rotation (seated)	Active ROM test of cervical rotation; distance displacement between suprasternal notch and the tragus of the right ear measured in cm.
Hip internal rotation	Active ROM test of bilateral internal rotation in a seated position; distance between medial malleoli measure in cm.
Hip abduction	Active ROM test of hip abduction in standing position; angle between level of ASIS and femur measured in degrees.
Shoulder flexion	Active ROM test of shoulder flexion; angle between torso and humerus measured in degrees.
5 × STS	Functional test of lower extremity strength by recording the time taken to complete five sit‐to‐stand repetitions.
Standing posture	Measurement of thoracolumbar spinal posture; physiotherapist measurement using flexicurve and surface topography using Kinect sensor both measured kyphosis index.

### 2.5 Laboratory research visit

The index of tests was instructed and measured by the physiotherapist, then during the same visit, the participants performed the tests for video recording under standardised instructions by the physiotherapist [16]. Self‐report and physical characteristic measures were also collected to compare characteristic differences between the two groups. Self‐reported disease‐specific questionnaires were collected: Bath AS Functional Index (BASFI), composed of 10 questions about functional limitation; the Bath AS Disease Activity Index (BASDAI), composed of six questions pertaining to fatigue, spinal pain, joint pain/swelling, areas of localised tenderness and morning stiffness and the Bath AS Patient Global score (BAS‐G) which asks about the person's well‐being over the past week and the past 6 months [16, 22–24]. Height and weight were measured.

### 2.6 Remote measurement collection

**FIGURE 1:**
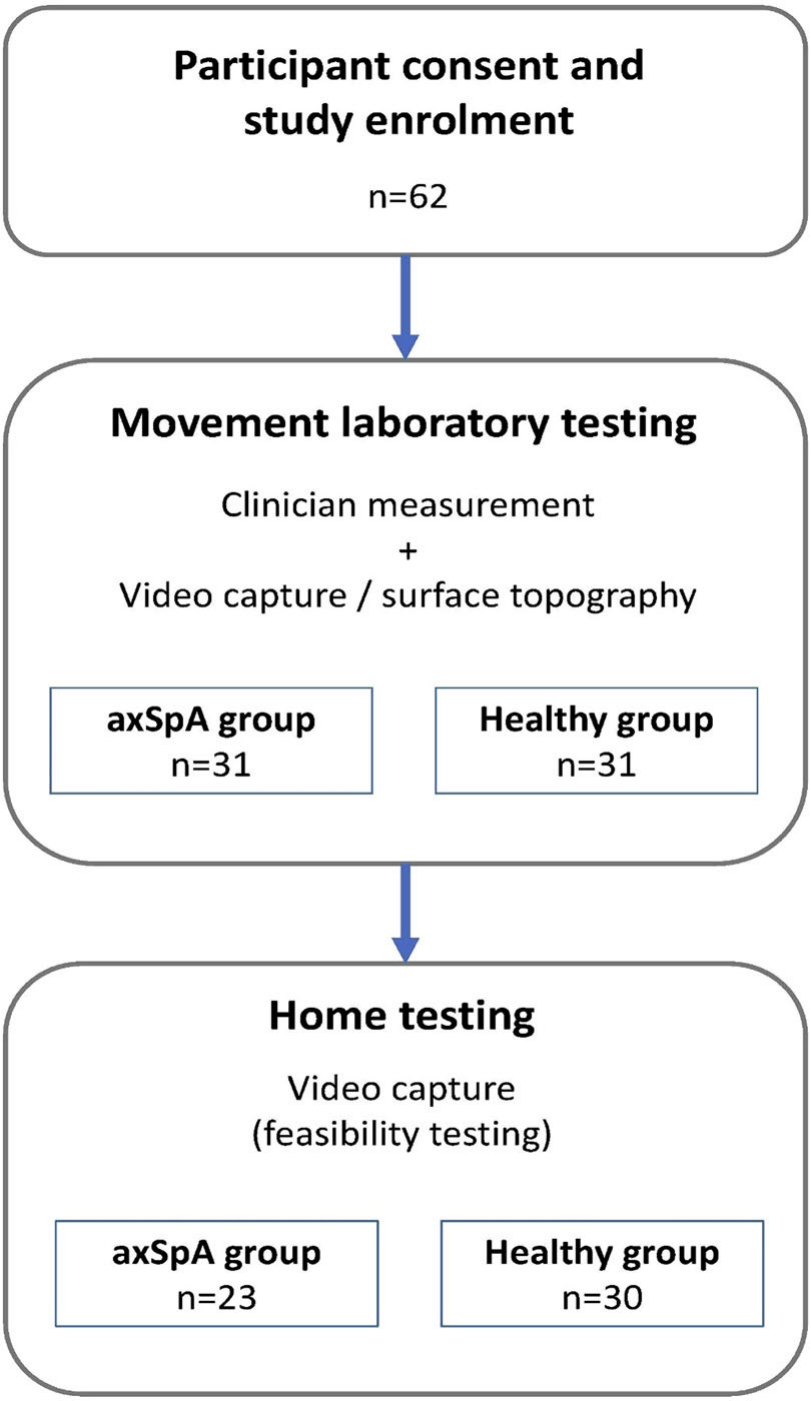
Study flowchart

After the laboratory research visit, the participant performed and captured video recordings of the same index of tests with standardised written instructions at their home using a personal smartphone camera, tablet camera or webcam (Figure 1) [16] within 1 week. Participants were given the option of a video support call with a physiotherapist during their home measurement.

### 2.7 Feasibility analysis

Completion rate and narrative participant feedback were collected to measure the feasibility of the CV‐aided system in terms of the practicality and acceptability of performing the tests in both home and laboratory settings. The completion rate of the outcome measures for both settings was recorded to help gain understanding of the internal and external barriers to implementation.

### 2.8 Cost‐consequence analysis

In order to analyse the cost‐benefits of an in‐person, physical clinical assessment and an automated, remote CV‐aided assessment, the direct costs and travel costs were calculated. Assumptions were made that an in‐person assessment would be carried out in a regional specialist service requiring an estimated travel of 30 miles roundtrip at £0.42/mile, and the associated carbon cost (average cost of CO_2_ emissions per car is 221.4 g/mile at £68/CO_2_) was calculated using the two methods to estimate the difference in cost per assessment. Additionally, associated benefits were compared in terms of measuring accuracy of CV compared to an assessment by a physiotherapist (in clinic).

### 2.9 Statistical analysis

All the data was coded anonymously. The Shapiro–Wilk test confirmed that all outcome measure data were normally distributed. Missing value analysis confirmed that missing data was randomly distributed and excluded for the comparisons. Descriptive statistics for each group were analysed and reported in the results with their mean (SD); independent sample *t*‐test was used to compare the group means between physical and self‐reported characteristics. Pearson's correlation analysis was used to compute the correlation between the two methods in each group, and Bland–Altman plot analysis used to estimate the agreement between methods within the axSpA cohort. Correlation coefficients 1.00 to 0.90 were interpreted as very strong, 0.89 to 0.70 as strong, 0.69 to 0.50 as moderate, 0.49 to 0.30 as weak and 0.29 to 0 as very weak [26]. Frequencies and percentages were used to summarise the feasibility data. *P* values <0.05 were considered statistically significant, and all tests were two‐tailed. Statistical analyses were performed using SPSS version 28 (IBM SPSS Statistics).

## 3 RESULTS

Sixty‐two participants (53% female) with a mean age of 45 (SD 14) years completed the study; there were 31 participants with axSpA (42% female, 54 (SD 13) years old) and 31 non‐back pain participants (65% female, 36 (SD 10) years old). The axSpA group had more functional limitations and higher disability compared to the non‐back pain group (Table 2). The axSpA group demonstrated more limited range of motion in the lumbar, shoulder and hip joints, and increased thoracic kyphosis and forward head posture compared to the non‐back pain group (Table 3).

**TABLE 2:** Participant characteristics based on gender and health condition

**Health condition**			**BMI (SD) [kg/m^2^]**	**Height (SD) [cm]**	**Weight (SD) [kg]**	**BASFI score (SD)** **[0–10; higher score = lower function]**	**BASDAI score (SD)** **[0–10; higher score = higher disability]**
Non‐back pain group	Male	Mean	27.57 (3.57)	179.5(4.7)	88.9 (12.1)	0.36 (0.51)	1.45(1.25)
		N	11	11	11	11	11
	Female	Mean	25.00 (3.48)	165.1 (7.8)	67.8 (8.1)	0.39 (0.54)	1.31 (1.16)
		N	20	20	20	20	20
	Total	Mean	25.91 (3.67)	170.2 (9.8)	75.3 (14.0)	0.38 (0.52)	1.36 (1.17)
		N	31	31	31	31	31
AxSpA group	Male	Mean	27.34 (3.24)	177.2 (7.9)	86.3 (15.5)	3.38 (2.11)	3.51 (1.71)
		N	18	18	18	18	18
	Female	Mean	27.37 (7.43)	159.6 (9.3)	68.6 (14.6)	3.76 (1.91)	3.49 (1.2)
		N	13	13	13	13	13
	Total	Mean	27.35 (5.30)	169.8 (12.2)	78.9 (17.3)	3.54 (2.01)	3.5 (1.69)
		N	31.00	31	31	31	31
Total	Male	Mean	27.43 (3.31)	178.1 (6.9)	87.3 (14.1)	2.24 (2.24)	2.73 (1.38)
		N	29	29	29	29	29
	Female	Mean	25.93 (5.41)	162.9 (8.7)	68.1 (10.9)	1.72 (2.09)	2.17 (1.76)
		N	33	33	33	33	33
	Total	Mean	26.63(4.58)	170.0 (11.0)	77.1 (15.7)	1.96 (2.16)	2.43 (1.80)
		N	62	62	62	62	62

**TABLE 3:** Movement and postural differences between groups measured by clinician assessment

**Test**	**AxSpA group** **[mean (SD)]**	**Non‐back pain group** **[mean (SD)]**	***p* value**
Seated cervical rotation (cm)	5.7 (1.4)	6.3 (0.8)	0.06
Lumbar forward flexion (cm)	33.5 (1.4)	18.7 (9.4)	<0.001
Hip internal rotation (cm)	36.4 (12.3)	47.4 (7.6)	<0.001
Shoulder flexion (°)			
Right shoulder	140.5 (21.6)	164.9 (18.7)	<0.001
Left shoulder	140.5 (26.3)	166.9 (18.8)	<0.001
Hip abduction (°)			
Right hip	30.6 (10.2)	44.0 (9.9)	<0.001
Left hip	29.8 (10.1)	44.6 (8.3)	<0.001
Lumbar side flexion (cm)			
Right side	12.9 (4.6)	20.3 (4.5)	<0.001
Left side	12.4 (4.9)	19.5 (3.1)	<0.001
Tragus‐to‐wall (cm)	16.7 (4.0)	13.2 (1.5)	<0.001
Thoracic kyphosis (index)	11.8 (3.9)	9.1 (3.6)	0.008
Lumbar lordosis (index)	10.4 (3.2)	12.27 (4.4)	0.08

### 3.1 Criterion validity testing for remote systems in lab setting

**FIGURE 2:**
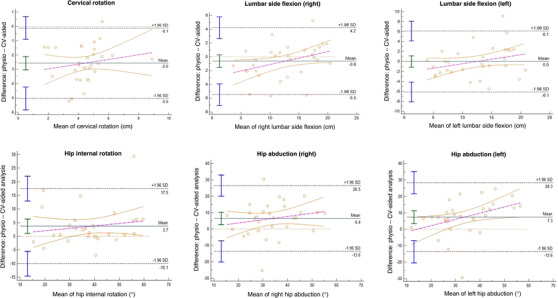
Bland–Altman plots for functional movements—agreement between CV‐aided analysis and physiotherapist

**FIGURE 3:**
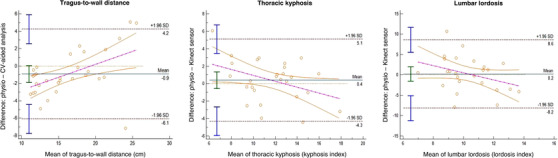
Bland–Altman plots for posture—agreement between remote technologies and physiotherapist

Cervical rotation measurement by the CV‐aided system was moderately correlated to a clinician assessment in the axSpA group and weakly correlated in the non‐back pain groups (Table 4); in the axSpA group, the CV‐aided system demonstrated a −2.6 cm bias compared to the reference physiotherapist measurement with a positive regression slope (Figure 2). Lumbar forward flexion and hip internal rotation were strongly correlated in both the axSpA and non‐back pain groups; both demonstrated a positive bias (+0.4 cm and +3.7 cm, respectively) with one outlier beyond the limits of agreement. Shoulder flexion and lumbar side flexion showed a strong to very strong correlation in the axSpA group and a moderate to weak correlation in the non‐back pain group. Shoulder flexion demonstrated a negative bias (right −3.0°, left −1.4°) with a slightly negative slope, and lumbar side flexion demonstrated minimal bias (right −0.6 cm, left 0 cm). Hip abduction was moderately correlated in axSpA group and demonstrated moderate to strong correlation in the non‐back pain group. Metrics for posture showed strong correlation for TWD and thoracic kyphosis measurement in the axSpA group, yet very weak (TWD) to moderate (kyphosis) correlation in the non‐back pain group; lumbar lordosis was not significantly correlated in either group (Table 4). All measurements showed agreement in the axSpA group with minimal bias (TWD −0.9, kyphosis +0.4, lordosis +0.2); TWD has a positive slope and kyphosis and lordosis have negative slopes, all with few outliers (Figure 3).

**TABLE 4:** Correlation between remote systems and clinician measurement for both groups

	AxSpA		Non‐back pain	
Test	*n*	*r*, *p* value	*n*	*r*, *p* value
Seated cervical rotation (cm)	31	0.649, <0.001	31	0.443, 0.013
Lumbar forward flexion (cm)	31	0.856, <0.001	31	0.858, <0.001
Hip internal rotation (cm)	30	0.854, <0.001	31	0.846, <0.001
Shoulder flexion (°)				
Right shoulder	30	0.787, <0.001	26	0.468, 0.016
Left shoulder	31	0.906, <0.001	30	0.533, 0.002
Hip abduction (°)				
Right hip	31	0.583, <0.001	31	0.683, <0.001
Left hip	31	0.643, <0.001	31	0.720, <0.001
Lumbar side flexion (cm)				
Right side	31	0.895, <0.001	31	0.476, 0.007
Left side	31	0.842, <0.001	31	0.655, <0.001
Tragus‐to‐wall (cm)	31	0.872, <0.001	31	0.194, 0.002
Thoracic kyphosis (index)	27	0.705, <0.001	28	0.553, 0.002
Lumbar lordosis (index)	25	−0.272, 0.183	28	0.239, 0.221

### 3.2 Feasibility of video capture in home setting

A total of 23 participants (74%) of 31 from the axSpA group uploaded their home‐recorded videos, and one participant did not use their calibration grid correctly during the videos. Based on these participants, the CV‐aided system produced an output in 84% for tragus‐to‐wall, 76% to 84% for shoulder flexion, 84% for lumbar forward flexion, 84% to 88% for lumbar side flexion, 84% to 88% for hip abduction, 88% for cervical rotation, 88% for hip internal rotation and 80% for the 5 × STS. Thirty non‐back pain participants (96%) out of 31 uploaded their videos; one participant did not utilise their calibration grid in the videos. Data from uploaded videos could be analysed for 71% for tragus‐to‐wall, 77% for shoulder flexion, 84% for lumbar forward flexion, 81% to 84% for lumbar side flexion, 87% for hip abduction, 84% for cervical rotation, 84% for hip internal rotation and 87% for 5×STS. There were no adverse events reported.

### 3.3 Cost‐consequence analysis

This cost analysis compared the CV‐aided system to the current clinical assessment costs that would incur in the UK's national health system. The results indicate that using this remote computer vision application for a physical movement assessment could save £64.70 for each participant per session with environmental, economic, and social benefits (Table 5). These analyses do not include other aspects of a comprehensive clinical assessment, including patient medical history, subjective reports and other routine medical testing, that comprise a typical clinic appointment.

**TABLE 5:** Comparison of costs between current clinical method and computer vision system

**Assessment**	**Cost**	**Benefit**
**Current clinical**	Physiotherapist Band 6 [24] £52.00 Travel £12.60 Carbon footprint £0.48 Total for 1 assessment £65.08	Face‐to‐face interaction preferred by minority of patients [recent survey Oxon rehab services] and for complex management.
**CV‐aided system**	Four assessments* £0.29 Total for one assessment £0.07 *Assuming 10% market of axSpA	No travel times Minimal carbon footprint Reduced carer pressure [driving] Self‐management Opportunity for more regular assessment Data can be securely transferred Expert physiotherapy for wider population, greater inclusion Saving clinical resource
**Total cost savings/benefit**	£64.70/assessment	Environmental Economic Social Reduced pressure on NHS

## 4 DISCUSSION

The study findings suggest that our camera remote measurement system has moderate to strong validity in a majority of functional and posture measurements compared to criterion clinical measurement in people with axSpA. The strongest correlational relationships were demonstrated in lumbar forward flexion, lumbar side flexion, shoulder flexion, hip internal rotation, tragus‐to‐wall and thoracic kyphosis, particularly in people with axSpA. The only test that showed no correlation and poor accuracy compared to the criterion method was lumbar lordosis, in both the axSpA and non‐back pain groups. In a home setting, our results suggest it is a feasible and cost‐effective method.

The two groups demonstrated expected clinical presentation differences, including higher BASDI and BASFI scores and a more restricted range of motion and hyperkyphosis in the axSpA group. The limited range of motion among the axSpA group in all functional movements tested demonstrates the broader use of this technology in clinical groups that fall outside the normal range of motion. In the end, the results did indicate varied correlative relationships between the axSpA and non‐back pain groups in several functional movements and postural tests, notably shoulder flexion, lumbar side flexion, tragus‐to‐wall and kyphosis. In both shoulder flexion and lumbar side flexion, the axSpA group had smaller ranges of motion compared to the non‐back pain group and stronger correlation (*r* = .787–.906) between the CV‐aided system and clinical measurement compared to the non‐back pain group (*r* = .468–.655). One reason for this discrepancy could be due to altered anatomical landmark visibility or increased trunk compensation in higher ranges of motion as were seen in the non‐back pain group. Posture measurements demonstrated similar incongruence; there was a stronger correlation in the axSpA group, who presented with more kyphotic and forward‐flexed posture compared to the non‐back pain group. This discrepancy could stem from less accurate and reliable measurement of smaller kyphosis curvature, which is one limitation of the tragus‐to‐wall test which has a floor effect [27]. The agreement trends between measures in the axSpA group should be noted as larger tragus‐to‐wall distances in the CV‐aided analysis corresponded to larger physiotherapist‐measured distances, and conversely, higher kyphosis angles corresponded to lower physiotherapist‐measured kyphosis angles.

The tests that did not demonstrate strong correlation were hip abduction, cervical rotation and lumbar lordosis posture. Hip abduction was adapted into a standing test to provide a more practical testing position for video recording compared to the BASMI hip mobility test, where the patient is lying on the ground and abducting both hips to their maximum range [23]. Although more practical to perform and standardise a camera set‐up, standing hip abduction has challenges that include both the participant performing it correctly and the landmarks needed for automation. Participants often compensate during standing hip abduction by either elevating their ipsilateral hip or externally rotating their hip. If the clinician does not correct the compensatory movements, it could cause an overestimation of the range. Similarly, the compensatory movements can cause an overestimation or inaccurate landmark identification by the CV algorithm. Cervical rotation in a seated position with a tape measure was also chosen from the EDASMI since the supine cervical rotation test from the BASMI presented challenges to camera positioning. The difficulty with frontal plane measurement of a rotational movement was demonstrated in the lack of a strong correlation between the CV‐aided system and clinician measurement, in both groups. Lastly, the lumbar lordosis postural alignment measured by surface topography using the Kinect sensor showed agreement, but no correlation and no significant difference between groups. This could be on account of the documented difficulty of measuring lumbar lordosis with surface measurement tools [28, 29], and clothing interference in some participants during the testing.

An important aspect of this study was the feasibility of the CV system in a home setting because of the potential for many benefits of remote testing. The first barrier for the participants was uploading the videos, which was less successful in the axSpA group (*n* = 8 missing) than the non‐back pain group (*n* = 1 missing). Developing a user‐friendly interface for uploading videos would lower the barrier for home use. Two other aspects of feasibility at home were the ability of participants to successfully record the correct movement and the quality of the videos for automated CV analysis. More than 70% of the recorded videos were useable. The reasons for non‐usable data were incorrect use of the calibration grid, camera movement and incompatible data format from one participant's smartphone. These issues could be addressed by improving instructions and calibration method.

Pragmatic use of this technology at home would be a key to helping people, with and without back pain, track and maintain functional movement, range of motion and posture with the option of remote clinician support. Not only does this remote system widen accessibility to specialists who may not be local, it is a cost‐efficient method and has many social and environmental benefits. It can benefit both patients and the health system in terms of time and opportunity. Furthermore, it can have a positive environmental impact by reducing the carbon footprint associated with each in‐person visit. The computer measurement is designed to assess only physical movements and therefore cannot replace the need for more interaction in virtual sessions or face‐to‐face appointments as there are many aspects of care that comprehensive assessments for axSpA contain. The value of remote technology assessments lies in the ability to monitor and track changes in physical movements, at increments unsustainable for in‐person visits in a system like the UK's national health system. Additionally, there is a place for these technologies to be an adjunct to face‐to‐face telemedicine, particularly useful for access to specialists. For the appropriate patient and need, it could result in a cost saving of £64 per assessment. While these results look specifically at people with axSpA, it can reasonably be generalised to similar long‐term musculoskeletal conditions.

### 4.1 Limitations

The limitations of this study include the relatively small sample size and the cross‐sectional method. While it was not possible for simultaneous measurement video recording and physiotherapist measurement since the physiotherapist would obstruct the anatomical reference points for CV‐aided analysis, the repetitions were performed within the same session under the same conditions. We recognise that there will still be error stemming from these methodological limitations. Future studies could possibly reduce this error by optimising time interval between tests. There would be benefit in future studies performing repeated testing to measure the sensitivity to change of these remote technologies, as well as potential sources of error associated with them. Lastly, a larger sample size of axSpA participants that included those with higher disease severity would be important to test to gain further insight into measurement agreement in the most restricted functional movement patterns.

## 5 CONCLUSIONS

Although not valid for clinical use in its current form, the remote technologies demonstrated moderate to strong correlation and agreement in most of the functional and postural tests measured in people with AxSpA. The results from testing the CV‐aided system in a home environment suggest it is a safe and feasible method, yet validity testing in this environment still needs to be performed.

## AUTHOR CONTRIBUTIONS

Erin Hannink: Methodology, Writing original draft. Maedeh Mansoubi: Formal analysis, Investigation, Methodology, Project administration, Validation, Writing–original draft. Neil Cronin: Methodology, Algorithm development. Benjamin Wilkins: Methodology, Revising the final draft. Ali A. Najafi: Data collection, Revising the final draft. Benjamin Waller: Contribution to study design, Revising the final draft. Helen Dawes: Methodology, Formal Analysis, Validation, Revising the final draft.

## CONFLICT OF INTEREST

Erin Hannink helped design the trial design as a paid consultant of Good Boost Wellbeing Ltd during the project but was not directly involved in data analysis. Neil Cronin developed the CV approach as a paid consultant of Good Boost Wellbeing Ltd during the project but was not directly involved in data collection. Benjamin Waller and Benjamin Wilkins were paid employees of Good Boost Wellbeing Ltd during the project but were not involved in data analysis or interpretation. All statistical comparisons between methods were performed independently by researchers at Oxford Brookes University and University of Exeter.

## Data Availability

Data will be made available upon request from the authors.

## References

[1] Hoy, D. , et al.: A systematic review of the global prevalence of low back pain. Arthritis Rheum. 64, 2028–2037 (2012) 22231424 10.1002/art.34347

[2] National Collaborating Centre for Primary Care : Low back pain: early management of persistent non‐specific low back pain. NICE Clinical Guidelines vol. 88 Preprint at (2009)

[3] Hamilton, L. , et al.: The prevalence of axial spondyloarthritis in the UK: A cross‐sectional cohort study. BMC Musculoskelet. Disord. 16, 1–5 (2015) 26690935 10.1186/s12891-015-0853-2 PMC4687290

[4] Yi, E. , Ahuja, A. , Rajput, T. , George, A.T. , Park, Y. : Clinical, economic, and humanistic burden associated with delayed diagnosis of axial spondyloarthritis: A systematic review. Rheumatol. Ther. 7, 65–87 (2020) 31965538 10.1007/s40744-020-00194-8 PMC7021861

[5] Fragoulis, G.E. , Siebert, S. : Treatment strategies in axial spondyloarthritis: what, when and how? Rheumatology 59, iv79–iv89 (2020) 33053192 10.1093/rheumatology/keaa435 PMC7566463

[6] Jenkinson, T.R. , et al.: Defining spinal mobility in ankylosing spondylitis (AS). The Bath AS Metrology Index. J. Rheumatol. 21, 1694–1698 (1994) 7799351

[7] Maksymowych, W.P. , et al.: Development and validation of the Edmonton Ankylosing Spondylitis Metrology Index. Arthritis Care Res. (Hoboken) 55, 575–582 (2006) 10.1002/art.22103 16874779

[8] Garrido‐Castro, J.L. , et al.: High reproducibility of an automated measurement of mobility for patients with axial spondyloarthritis. J. Rheumatol. 45, 1383–1388 (2018) 29907675 10.3899/jrheum.170941

[9] Garrido‐Castro, J.L. , et al.: Validation of a new objective index to measure spinal mobility: The University of Cordoba Ankylosing Spondylitis Metrology Index (UCOASMI). Rheumatol. Int. 34, 401–406 (2014) 24356712 10.1007/s00296-013-2917-7

[10] Concepción Aranda‐Valera, I. , et al.: Measuring spinal mobility using an inertial measurement unit system: A validation study in axial spondyloarthritis. Diagnostics (Basel) 10, 426 (2020) 32599741 10.3390/diagnostics10060426 PMC7344521

[11] Franco, L. , Sengupta, R. , Wade, L. , Cazzola, D. : A novel IMU‐based clinical assessment protocol for Axial Spondyloarthritis: A protocol validation study. Peer J. 9, 1–29 (2021) 10.7717/peerj.10623 PMC7845531 33569248

[12] Hellsten, T. , Karlsson, J. , Shamsuzzaman, M. , Pulkkis, G. : The potential of computer vision‐based marker‐less human motion analysis for rehabilitation. Rehabil. Process Outcome 10, 117957272110223 (2021) 10.1177/11795727211022330 PMC8492027 34987303

[13] Mokkink, L.B. , et al.: COSMIN study design checklist for Patient‐reported outcome measurement instruments. COSMIN www.cosmin.nl/wp‐content/uploads/COSMIN‐study‐designing‐checklist_final.pdf (2019)

[14] von Elm, E. , et al.: Strengthening the reporting of observational studies in epidemiology (STROBE) in the international journal of medical students. BMJ 335, 806–808 (2007) 17947786 10.1136/bmj.39335.541782.AD PMC2034723

[15] Cao, Z. , Hidalgo, G. , Simon, T. , Wei, S.‐E. , Sheikh, Y. : OpenPose: Realtime multi‐person 2D Pose estimation using part affinity fields. IEEE Trans. Pattern Anal. Mach. Intell. 43, 172–186 (2021) 31331883 10.1109/TPAMI.2019.2929257

[16] Hannink, E. , Mansoubi, M. , Cronin, N.J. , Waller, B. , Dawes, H. : Computer‐vision aided functional movement measurement in people with and without axial spondyloarthritis – validation and feasibility study protocol. OSF Preprints (2021). 10.31219/osf.io/hsv7p

[17] Bradski, G. : The OpenCV library. Dr. Dobb's J. Softw. Tools 120, (2000)

[18] Hannink, E. , Shannon, T. , Barker, K.L. , Dawes, H. : The reliability and reproducibility of sagittal spinal curvature measurement using the Microsoft Kinect V2. J. Back Musculoskelet. Rehabil. 33, 295–301 (2020) 31356192 10.3233/BMR-191554

[19] Perrotta, F.M. , Musto, A. , Lubrano, E. : New insights in physical therapy and rehabilitation in axial spondyloarthritis: A review. Rheumatol. Ther. 6, 479–486 (2019) 31410786 10.1007/s40744-019-00170-x PMC6858478

[20] Greendale, G.A. , Nili, N.S. , Huang, M.H. , Seeger, L. , Karlamangla, A.S. : The reliability and validity of three non‐radiological measures of thoracic kyphosis and their relations to the standing radiological Cobb angle. Osteoporos. Int. 22, 1897–1905 (2011) 20938766 10.1007/s00198-010-1422-z PMC3092935

[21] de Oliveira, T.S. , et al.: Validity and reproducibility of the measurements obtained using the flexicurve instrument to evaluate the angles of thoracic and lumbar curvatures of the spine in the sagittal plane. Rehabil. Res. Pract. 2012, 1–9 (2012) 10.1155/2012/186156 PMC3348664 22619723

[22] Jones, S.D. , Steiner, A. , Garrett, S.L. , Calin, A. : The bath ankylosing spondylitis patient global score (BAS‐G). Br. J. Rheumatol. 35, 66–71 (1996) 8624626 10.1093/rheumatology/35.1.66

[23] Garrett, S. , et al.: A new approach to defining disease status in ankylosing spondylitis: The bath ankylosing spondylitis disease activity index. J. Rheumatol. 21, 2286–2291 (1994) 7699630

[24] Calin, A. , et al.: A new approach to defining functional ability in ankylosing spondylitis: The development of the bath ankylosing spondylitis functional index. J. Rheumatol. 21, 2281–2285 (1994) 7699629

[25] Esser, P. , Dawes, H. , Collett, J. , Feltham, M.G. , Howells, K. : Assessment of spatio‐temporal gait parameters using inertial measurement units in neurological populations. Gait Posture 34, 558–560 (2011) 21764583 10.1016/j.gaitpost.2011.06.018

[26] Hinkle, D.E. , Wiersma, W. , Jurs, S. G. : Applied Statistics for the Behavioral Sciences. Boston, MA: Houghton Mifflin Company. (2003)

[27] Ogdie, A. , et al.: Measuring outcomes in axial spondyloarthritis. Arthritis Care Res. (Hoboken) 72, 47–71 (2020) 33091248 10.1002/acr.24266

[28] Sedrez, J.A. , Candotti, C.T. , Furlanetto, T.S. , Loss, F. : Non‐invasive postural assessment of the spine in the sagittal plane : a systematic review. Motricidade 12, 140–154 (2016)

[29] Krott, N.L. , Wild, M. , Betsch, M. : Meta‐analysis of the validity and reliability of rasterstereographic measurements of spinal posture. Eur. Spine J. 29, 2392–2401 (2020) 10.1007/s00586-020-06402-x 32277336

